# The Beneficial Role of Sunitinib in Tumor Immune Surveillance by Regulating Tumor PD‐L1

**DOI:** 10.1002/advs.202001596

**Published:** 2020-11-27

**Authors:** Hui Li, Xinwei Kuang, Long Liang, Youqiong Ye, YongChang Zhang, Jialu Li, Fangyu Ma, Juan Tao, Guang Lei, Shuang Zhao, Juan Su, Nong Yang, Cong Peng, Xiaowei Xu, Mien‐Chie Hung, Leng Han, Hong Liu, Jing Liu, Xiang Chen

**Affiliations:** ^1^ Department of Dermatology Xiangya Hospital Central South University Changsha Hunan 410008 China; ^2^ Molecular Biology Research Center Center for Medical Genetics Hunan Province Key Laboratory of Basic and Applied Hematology School of Life Sciences Central South University Changsha 410078 China; ^3^ Molecular Science and Biomedicine Laboratory State Key Laboratory for Chemo/Biosensing and Chemometrics College of Biology College of Chemistry and Chemical Engineering Collaborative Innovation Center for Chemistry and Molecular Medicine Hunan University Changsha Hunan 410082 China; ^4^ Hunan Key Laboratory of Skin Cancer and Psoriasis Changsha Hunan 410008 China; ^5^ Hunan Engineering Research Center of Skin Health and Disease Changsha Hunan 410008 China; ^6^ Xiangya Clinical Research Center for Cancer Immunotherapy Central South University Changsha Hunan 410008 China; ^7^ Shanghai Institute of Immunology Department of Immunology and Microbiology Center for Single‐Cell Omics State Key Laboratory of Oncogenes and Related Genes Shanghai Jiao Tong University School of Medicine Shanghai 200025 China; ^8^ Department of Biochemistry and Molecular Biology The University of Texas Health Science Center at Houston McGovern Medical School Houston TX 77030 USA; ^9^ Department of Medical Oncology Lung Cancer and Gastrointestinal Unit Hunan Cancer Hospital/The Affiliated Cancer Hospital of Xiangya School of Medicine Central South University Changsha Hunan 410013 China; ^10^ Department of Biostatistics HuaJia Biomedical Intelligence ShenZhen 518054 China; ^11^ Department of Health Management Center Xiangya Hospital Central South University Changsha Hunan 410008 China; ^12^ Department of Dermatology Affiliated Union Hospital Tongji Medical College Huazhong University of Science and Technology Wuhan 430022 China; ^13^ Department of Radiation Oncology Hunan Cancer Hospital and The Affiliated Cancer Hospital of Xiangya School of Medicine Central South University Changsha Hunan 410013 China; ^14^ Department of Pathology and Laboratory Medicine Perelman School of Medicine 42 University of Pennsylvania Philadelphia PA 19104 USA; ^15^ Graduate Institute of Biomedical Sciences and Center for Molecular Medicine China 44 Medical University Taichung 404 Taiwan; ^16^ Department of Biotechnology Asia University Taichung 413 Taiwan; ^17^ Research Center of Molecular Metabolomics Xiangya Hospital Central South University Changsha Hunan 410008 China

**Keywords:** immune surveillance, p62, PD‐L1, selective autophagy, sunitinib

## Abstract

Immune checkpoints blockades have shown promising clinical effects in various malignancies, but the overall response rate is low. Here, the immune features are comprehensively characterized in >10 000 cancer patients from The Cancer Genome Atlas and significantly positive correlations are observed between targets of Sunitinib and inhibitory immune checkpoints and suppressive immune cells. It is further confirmed that Sunitinib treatment increases the antitumor immunity in a phase III trial. Mechanistically, it is discovered that Sunitinib regulates the stability of tumor PD‐L1 via p62, that p62 can bind to PD‐L1 and specifically promote its translocation into autophagic lysosome for degradation. Preclinically, Sunitinib shows a synergistic antitumor effect with cytotoxic T‐lymphocyte‐associated protein 4 (CTLA‐4) monoclonal antibody (mAb) in melanoma and nonsmall cell lung cancer (NSCLC) immune competent mice by promoting the tumor‐infiltrating lymphocytes activity. Clinically, a higher PD‐L1 level but a lower p62 level in the tumor region of responders as compared to those of nonresponders among anti‐PD‐1‐treated NSCLC patients is observed. Taken together, by utilizing rigorous computational analysis, functional characterization in vitro and in vivo, and neoadjuvent clinical trial, a novel molecular mechanism is revealed regarding the regulation of PD‐L1 via p62, thus providing a novel therapeutic strategy by the combination treatment of CTLA‐4 with Sunitinib.

## Graphical abstract

This study observes positive correlations between targets of Sunitinib and inhibitory immune checkpoints and suppressive immune cells, reveals the role of Sunitinib in immune surveillance by promoting p62‐mediated PD‐L1 degradation via autophagy, and provides a novel therapeutic strategy by combination treatment of Sunitinib and cytotoxic T‐lymphocyte‐associated protein 4 monoclonal antibody.

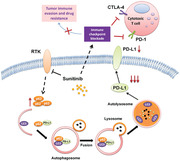

## 1 Introduction

Immune checkpoints blockades targeting the interaction between programmed cell death protein 1 (PD‐1) and its ligand programmed death ligand 1 (PD‐L1; also known as B7‐H1 and CD274) have shown promising clinical effects in various malignancies including metastatic melanoma and nonsmall cell lung cancer (NSCLC).^[^ ^1^, ^2^, ^3^, ^4^, ^5^, ^6^ ^]^ However, the overall response rate is less than 40% in general.^[^ ^7^, ^8^, ^9^ ^]^ Recent studies have shown that the tumor PD‐L1 level was regarded as a predicting biomarker for assessing the clinical response to anti‐PD‐1/PD‐L1 therapy. Thus, it is critical to understand the molecular mechanism regarding the regulation of tumor PD‐L1, which may provide novel strategy to enhance the therapeutic efficacy.

Sunitinib, a multitargeted receptor tyrosine kinase (RTK) inhibitor approved by the food and drug administration (FDA) at 2006,^[^ ^10^ ^]^ is currently utilized as a standard of care for both clear cell renal cell carcinoma (ccRCC) and gastrointestinal stromal tumors.^[^ ^11^ ^]^ However, the role of Sunitinib in tumor immune surveillance and whether it is involved in the regulation of PD‐L1 is completely unknown. Previous studies have reported that the tumor PD‐L1 level can be regulated by gene amplification, epigenetic modifiers (e.g., BRD4, MLL1), microRNAs (e.g., miR‐200, miR570, and miR‐138‐5p), transcriptional level (e.g., interferon gamma (IFN‐*γ*) janus kinase‐signal transducer and activator of transcription (JAK‐STAT), nuclear factor kappa‐B (NF‐*κ*B), hypoxia inducible factor‐1α (HIF‐1*α*), EGFR, Hippo signaling pathway), and post‐translational modification (e.g., COP9 signalosome 5 (CSN5) and adenosine 5‘‐monophosphate (AMP)‐activated protein kinase (AMPK).^[^ ^12^, ^13^, ^14^, ^15^, ^16^, ^17^ ^]^ Our results showed that Sunitinib treatment decreased PD‐L1 protein levels in a dose‐dependent manner reflected by western blotting, but did not affect PD‐L1 mRNA levels, indicating that Sunitinib post‐transcriptional regulated PD‐L1 level. Further pathway analysis suggested that Sunitinib controlled PD‐L1 protein stability via autophagy, which is a highly conserved pathway for the degradation and recycling of cytoplasmic components in under‐stress conditions.^[^ ^18^ ^]^

The process of autophagy can be either nonselective or selective, where highly selective one involves in the turnover of damaged organelles, removal of protein aggregates, and elimination of intracellular pathogens.^[^ ^19^ ^]^ The cargo recognition and trafficking in the selective process depend on the cargo receptors like SQSTM1/p62.^[^ ^20^, ^21^ ^]^ The p62 utilizes its C‐terminal ubiquitin‐associated domain to interact with the ubiquitinated cargoes as well as the microtubule associated protein 1 light chain 3 (LC3) in the inner membrane of the phagophore. Furthermore, the PB1 domain of p62 mediates homopolymerization of p62 and facilitates its interaction with the cargo.^[^ ^22^ ^]^ There are a series of cargo receptors that act as a bridge between the ubiquitinated proteins or organelles and gamma‐aminobutyric acid receptor‐associated protein (LC3/GABARAP) family members in the core autophagy machinery.^[^ ^23^ ^]^ The dysfunction of selective autophagy is related to the development of cancer, aging and other diseases.^[^ ^24^, ^25^, ^26^, ^27^ ^]^

In this study, by utilizing computational analysis, functional characterization in vitro and in vivo, and neoadjuvent clinical trial, we revealed a novel molecular mechanism regarding the regulation of PD‐L1 via p62 and provided a novel therapeutic strategy by the combination treatment of cytotoxic T‐lymphocyte‐associated protein 4 (CTLA‐4) with Sunitinib.

## 2 Results

### 2.1 Associations between Sunitinib Targets and Immune Suppressive Cells and Inhibitory Immune Checkpoints

**Figure 1:**
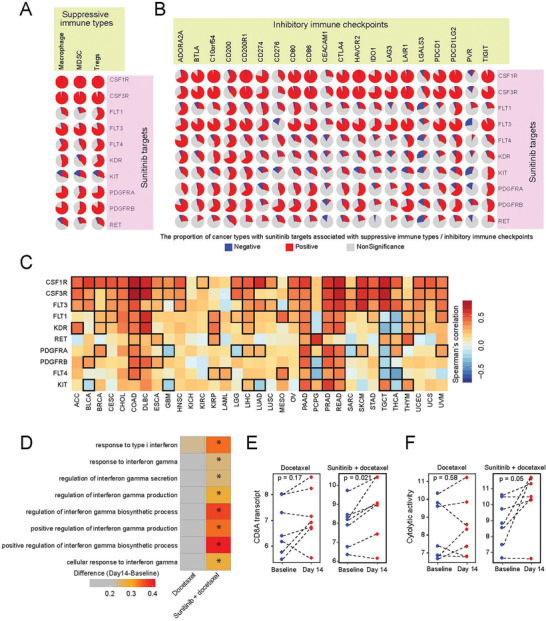
Sunitinib and its targets play a crucial role in the regulation of tumor immunity. A,B) The proportion of cancer types with positive (Rs > 0.3 and FDR < 0.05, in red), negative (Rs < −0.3 and FDR < 0.05, in blue), or nonsignificant (in gray) correlation for A) the relative abundance of suppressive immune cell types or B) inhibitory immune checkpoints and Sunitinib targets in 33 cancer types (*n* = 10 205). C) The Spearman's correlation of mRNA expression between PD‐L1 and Sunitinib targets in cancers (*n* = 10 205). Black box indicates the significant correlations (|Rs| > 0.3 and FDR < 0.05). D) Docetaxel‐ and sunitinib + docetaxel‐induced changes from baseline in the GSVA of IFN‐*γ* related GO terms, E) CD8A transcript, and F) cytolytic activity to 14 d after treatment in cancer patients (*n* = 14).

To explore whether Sunitinib is involved in the antitumor immunity, we systematically examined the expression of immune features and the Sunitinib targets through Spearman correlation in The Cancer Genome Atlas (TCGA) corhort.^[^ ^28^ ^]^ We found that most of the Sunitinib targets were significantly positive correlation for the relative abundance of suppressive immune cell types, including tumor associated macrophage, myeloid‐derived suppressor cells (MDSC), and regulatory T cells (Tregs) (**Figure** **1**A). For example, colony stimulating factor 1 receptor (CSF1R), a Sunitinib target, correlated with macrophage in 33 cancers (median Rs = 0.80), MDSC in 33 cancers (median Rs = 0.84), and Tregs in 33 cancers (median Rs = 0.83). Sunitinib targets were correlated with inhibitory immune checkpoints in most cancer types from TCGA corhort (Figure 1B). Among these, PD‐L1 were significantly positive correlated with most of the Sunitinib targets in 33 cancer types (Figure 1C). For example, mRNA expression of PD‐L1 is significantly correlated with CSF1R in 26 cancers (median Rs = 0.48). Our analysis demonstrated a strong correlation between Sunitinib and suppressive immune features, including PD‐L1. To further confirm the potential role of Sunitinib in antitumor immunity, we analyzed 14 patients of a phase III trial of metastatic breast cancer treated with Sunitinib combined with docetaxel versus docetaxel alone.^[^ ^29^ ^]^ We observed a significant increase for IFN‐*γ* related gene ontology (GO) terms (Figure 1D), CD8A transcript (Figure 1E) and cytolytic activity (CYT),^[^ ^30^ ^]^ a proxy to reflect the capacity of T cells to kill cancer cells (Figure 1F). Sunitinib combined with Docetaxel treatment significantly activated the IFN‐*γ* related signaling pathway and upregulated the CD8A transcript and cytolytic activity, suggesting that Sunitinib treatment associated with T cell infiltration and activity.

### 2.2 Sunitinib Treatment Enhanced CTL Activity Associated with Decrease of Tumor PD‐L1

**Figure 2:**
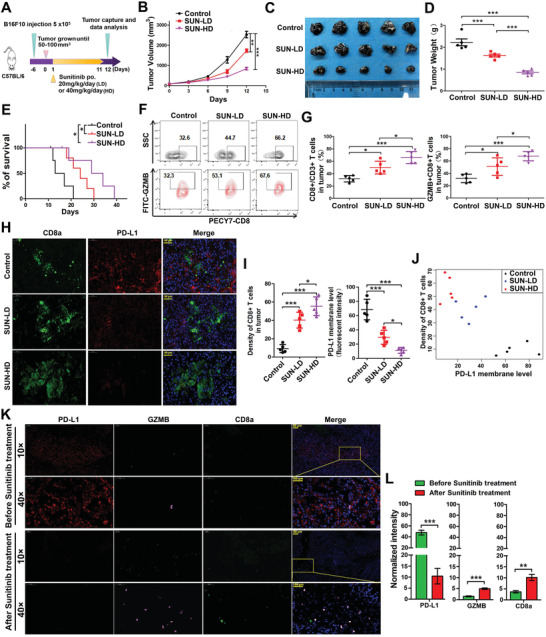
Sunitinib enhances CTL activity by reduction of tumor cell PD‐L1. A) Mouse melanoma cell line B16F10 cells were injected into mice (*n* = 10 mice per group) on day 0, and low dose (LD, 20 mg kg^−1^) or high dose (HD, 40 mg kg^−1^) of Sunitinib were administered as the chart indicated. B) Tumor volume was measured on the indicated different time points. Data represent mean ± SD. Tumor growth analysis for Figure 1C through pairwise comparisons at each time in Table S1 in the Supporting Information. C) Representative images of tumors after different does Sunitinib treatment at the end of time point in the B16F10 tumor burden mouse model. D) Mice were sacrificed at day 12 after Sunitinib treatment, Tumor weight was measured on the Day 12. Data represent mean ± SD, *p < 0.05, ***p < 0.001. E) Kaplan–Meier survival curves for mice bearing B16F10 tumors following treatment with different does of Sunitinib. Significance was determined by the Gehan–Breslow–Wilcoxon test (*n* = 10 mice per group), Data represent mean ± SD, *p < 0.05,***p < 0.001. F) Representative profiles of flow cytometry‐based detection of the CD8 (CTL marker) and granzyme B (GZMB) the marker of activity of the T cell in B16F10 tumor mass form the different treatment groups. G) The quantification the CD8^+^/CD3^+^ and CD8^+^ GZMB^+^ CTL cell percentage in the tumor mass from different groups of treatment (*n* = 5 mice per group). Data represent mean ± SD, *p < 0.05,***p < 0.001. H) Representative image of immunofluorescence staining for immunostaining of CD8 and PD‐L1 in the B16F10 tumor mass. The fluorescence of green (FITC) indicated the CD8 and red (Cy3) indicated the PD‐L1. Blue (DAPI) indicated the nucleus. Scale bar, 50 µm in inset. I) The quantification percentage of the CD8^+^ and PD‐L1^+^ cell in the tumor mass from different groups of treatment (*n* = 5 mice per group). Data represent mean ± SD, *p < 0.05,***p < 0.001. J) Scatter plot of PD‐L1 membrane level and density of CD8^+^ cells using data generated from Figure 1H,I. The Pearson correlation coefficient is −0.845, indicating a strong negative correlation. K) Representative image of immunofluorescence staining for immunostaining of CD8 and PD‐L1 in tumor sample before and after Sunitinib treatment. The fluorescence of green (FITC) indicated the GZMB, pink (Cy5) indicated the CD8, and red (Cy3) indicated the PD‐L1. Blue (DAPI) indicated the nucleus. Scale bar, 50 µm in inset. L) The quantification of normalized fluorescence intensity of PD‐L1, GZMB, and CD8a per sample in tumor mass from before and after Sunitinib treatment patients are expressed as mean ± SD, six views per sample, **p < 0.01, ***p < 0.001.

To investigate the antitumor immunity of Sunitinib, we treated melanoma B16F10 tumor‐bearing immune competent mouse with Sunitinib (**Figure** **2**A). Sunitinib treatment significantly reduced mouse tumor burden (mean tumor size: control vs Sunitinib (SUN)‐low dose (LD) (2533 vs 1733 mm^3^, *p* < 0.01); control vs SUN‐high dose (HD) (2533 vs 844 mm^3^, *p* < 0.001); by one‐way analysis of variance (ANOVA), Dunnett's multiple comparison test; Figure 2B–D), and substantially extended the overall survival (OS) time (median survival: control vs SUN‐LD (15 vs 24 d; *p* < 0.05); control vs SUN‐HD (15 vs 31.5 d; *p* < 0.05); log‐rank test; Figure 2E). The antitumor effect of such treatment increased with time as compared to that of the control (Table S1, Supporting Information). More importantly, administration of Sunitinib did not result in significant body weight change (Figure S2A, Supporting Information), suggesting the limited toxicity of Sunitinib treatments in tumor‐bearing mice. The immunity‐based cancer cell elimination depends primarily on the activated CD8^+^ T cells, we therefore investigated infiltrated lymphocytes in the tumor region of Sunitinib treated mice. The number of tumor infiltrated activated CD8^+^ T cell population (granzyme B, GZMB^+^/CD8^+^) significantly increased in Sunitinib treated mice compared to the vehicle group (from 32.3% to 53.1% (SUN‐LD), *p* < 0.05; or to 67.6% (SUN‐HD), *p* < 0.05; Figure 2F–G and Figure S2B, Supporting Information). Since the activity of CD8^+^ T cell is mainly controlled by immune checkpoints, we hypothesized that Sunitinib might affect the activity of CTLs by regulating the level of immune checkpoints. As showed in Figure 2H–J, Sunitinib can increase the abundance of CTL but decrease the PD‐L1 level, and the PD‐L1 expression level has a strong negative correlation (Rs = 0.713; *p* < 0.001) with the CTL population size. Such relationship was also observed in renal cell carcinoma (RCC) patient samples, where the PD‐L1 decreases with the induction of GZMB^+^CD8^+^ T cell after the patients received Sunitinib treatment (Figure 2K–L). These results suggest that the antitumor CTL activity enhanced by Sunitinib may function via its regulation of PD‐L1.

### 2.3 Sunitinib Regulates Tumor PD‐L1 via p62‐Mediated Selective Autophagy

**Figure 3:**
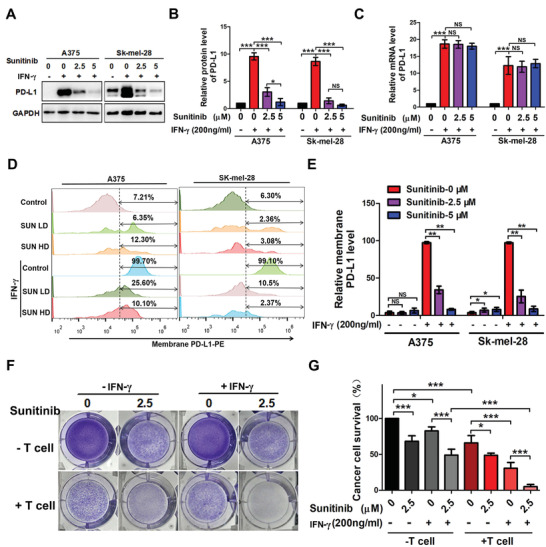
Sunitinib regulates the stability of the PD‐L1. A) Representative Western blot analysis of A375 and SK‐MEL‐28 cells treated with increasing concentrations of Sunitinib (2.5–5 µmol) for 24 h under IFN‐*γ* exposure. B) Bar diagram presenting the quantitative analysis of PD‐L1 protein expression data from (A). The plot was generated from three independent experiments and showed as means ± SD, *p < 0.05, ***p < 0.001. Linear regression model fitting results showed that the regression coefficients of Sunitinib dose in A375 and SK‐MEL‐28 were −1.67 and −1.60, respectively. Both coefficients are significantly different from zero with a *p*‐value <0.001, indicating a significant dose effect. C) Bar graph presentation of PD‐L1 mRNA levels as determined by qRT‐PCR of increasing concentrations of Sunitinib treatment of two cell lines for 24 h under IFN‐*γ* exposure. The plot was generated from three independent experiments and showed as means ± SD, *p < 0.05, ***p < 0.001. D) Representative profiles of flow cytometry analysis of membrane PD‐L1 expression by flow‐cytometric analysis after increasing concentrations of Sunitinib (2.5–5 µmol) treated A375 and SK‐MEL‐28 cells for 24 h under IFN‐*γ* exposure. E) The quantitative analysis of flow cytometry analysis of membrane PD‐L1 expression by flow‐cytometric analysis after increasing concentrations of Sunitinib (2.5–5 µmol) treated A375 and SK‐MEL‐28 cells for 24 h under IFN‐*γ* exposure. F) A375 cells cocultured with activated T cells for 48 h with or without Sunitinib (5 µmol) were subjected to crystal violet staining. The tumor cell to T cell ratio, 1:3. G) Bar diagram presenting the quantitative analysis of cancer cell survive rate from (F). The plot was generated from three independent experiments and showed as means ± SD, *p < 0.05, ***p < 0.001.

To further assess whether Sunitinib regulates tumor PD‐L1 level, melanoma cells were pretreated by IFN‐*γ* to induce PD‐L1 level and followed by treatment with or without Sunitinib. The protein levels of PD‐L1 significantly decreased in a Sunitinib dose‐dependent manner as shown by Western blotting analysis (**Figure** **3**A,B), but the mRNA levels of PD‐L1 were not affected by Sunitinib treatment as measured by quantitative realtime‐polymerase chain reaction (qRT‐PCR) (Figure 3C). Flow cytometry analysis also showed IFN‐*γ*‐induced PD‐L1 membrane level decreased in a dose‐dependent manner after the exposure of melanoma cells (in A375 from 99.7% to 10.1% and SK‐MEL‐28 from 99.1% to 2.37%; Figure 3D,E) or A549 cells (from 87.4% to 16.5%; Figure S3A,B, Supporting Information) to Sunitinib. Furthermore, T cell killing assay was performed to test the effect of Sunitinib‐associated expression level change of tumor PD‐L1 on the CTL activity. As expected, Sunitinib significantly enhanced the T cell killing ability (Figure 3F,G). Therefore, these results suggest that Sunitinib regulates the PD‐L1 expression via protein‐level modifications, rather than at the transcriptional level.

**Figure 4:**
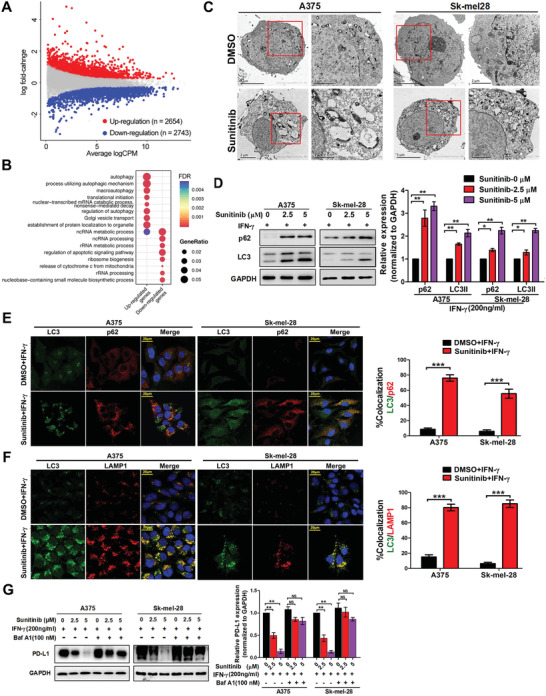
Sunitinib downregulates PD‐L1 via autophagic degradation. A) Summary of the distribution of differentially expressed genes (DE genes) identified from RNA sequencing data, which was generated from the A375 cells with or without Sunitinib treatment. The average logCPM (count per million) represented the gene expression level. The color represents differentially expressed genes (fold change >1.3, FDR < 0.05, see the Experimental Section; red: upregulation, blue: downregulation, gray: nonsignificant change). B) Top eight enriched GO biological processes pathways for genes upregulated or downregulated in A375 melanoma cell lines treated with Sunitinib. C) Representative image of the detection of autophagy using a transmission electron microscope. A375 or SK‐MEL‐28 cells were treated with 5 µmol Sunitinib for 24 h or dimethyl sulfoxide (DMSO) treated. The right side image indicates: autolysosome‐like structures. Bar, 2 µm. D) Representative image and quantification of Western blot analysis of p62 and LC3 in A375 and SK‐MEL‐28 cells after treatment with indicated doses of Sunitinib. GAPDH was used as loading control (left). Bar graphs are quantified results of three independent experiments and showed as means ± SD, *p < 0.05, **p < 0.01 (right). E,F) Representative image and colocalization coefficiency of immunofluorescence staining in A375 and SK‐MEL‐28 cells for immunostaining of LC3 (green FITC) with red (Cy3) staining of E) p62 or F) LAMP1, nuclear was indicated by DAPI (blue). Scale bar, 20 µm in inset. Data are representatives of three independent assays. The LC3 and p62 or LAMP1 colocalization coefficiency is expressed as mean ± standard error of mean (SEM), *n* = ≈30–50 cells of three independent experiments. G) Representative image and quantification of western blot analysis of PD‐L1 in A375 and SK‐MEL‐28 cells after treatment with indicated doses of Sunitinib with or without BafA1 (the inhibitor of autophagy) under the IFN‐*γ* exposure for 24 h. GAPDH was used as loading control (left). Bar graphs was quantified results of three independent experiments expressed as the mean ± SEM, *p < 0.05, **p < 0.01 compared to the control (right).

To explore the molecular mechanism of Sunitinib‐mediated regulation of PD‐L1, we performed RNA‐seq to identify the signaling pathways altered by Sunitinib in A375 cells (Table S2, Supporting Information). We found 2654 significantly upregulated genes and 2743 downregulated genes upon treatment with Sunitinib (**Figure** **4**A, see the Experimental Section). In particular, the upregulated genes were significantly enriched in autophagy related genes (Figure 4B). We further performed gene set enrichment analysis (GSEA) and showed that autophagy related genes are significantly upregulated in cells with Sunitinib treatment (Figure S4, Supporting Information). Based on aforementioned evidence that Sunitinib regulates tumor PD‐L1 at the protein level, we tested whether this regulation is dependent on autophagy. First, under electron microscopy, we showed that the number of autophagosomes and lysosomes were increased in two Sunitinib‐treated melanoma cells (Figure 4C). Second, two autophagy‐related markers, the cargo receptor p62 and the autophagosome membrane marker LC3II, increased significantly after Sunitinib treatment (Figure 4D). The colocalization between LC3II and p62 or lysosome marker lysosome‐associated membrane protein 1 (LAMP1) was enhanced after Sunitinib treatment (Figure 4E,F), suggesting that Sunitinib is able to induce autophagic flux. Third, when combining autophagy inhibitor bafilomycin A1 (Baf A1) with Sunitinib for the treatment of melanoma cells, we found that Baf A1 can significantly rescue the Sunitinib‐associated decrease of PD‐L1 level (Figure 4G).

**Figure 5:**
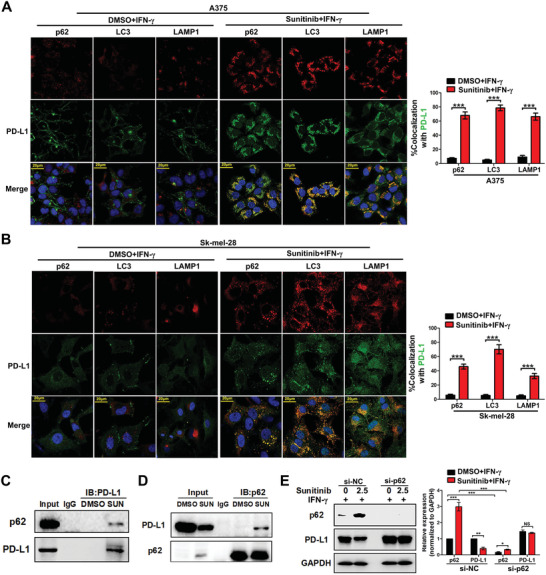
Sunitinib regulates PD‐L1 via p62 dependent selective autophagy. A,B) Representative image (left) and colocalization coefficiency (right) of immunofluorescence staining in A) A375 and B) SK‐MEL‐28 cells for immunostaining of PD‐L1 (green FITC) with red (Cy3) staining of LC3, p62 or LAMP1, nuclear was indicated by DAPI (blue). Scale bar, 20 µm in inset. Data are representatives of three independent assays. The PD‐L1 and LC3, p62 or LAMP1 colocalization coefficiency is expressed as mean ± SEM, *n* = ≈30–50 cells of three independent experiments. C) Representative Western blot analysis of p62 after immunoprecipitation of endogenous PD‐L1 as indicated treatment from A375 cells. D) Representative Western blot analysis of PD‐L1 after immunoprecipitation of endogenous p62 as indicated treatment from A375 cells. E) Representative Western blot analysis of knockdown p62 in A375 cells treated with or without Sunitinib (2.5 µmol) for 24 h under IFN‐*γ* exposure (left). Bar graphs were quantified results of three independent experiments expressed as the mean ± SEM, *p < 0.05, **p < 0.01 compared to the control (right).

We then focused the scope of autophagy process on p62 gene. As an important adapter of autophagy, p62 has been found to be involved in both nonselective and selective autophagy, but the increase of p62 usually indicates a selective process. The p62 can bind to the substrate of LC3 and translocate to autophasome for degradation through fusion with the lysosome.^[^ ^31^ ^]^ Here, we found in the RNA sequencing data that the selective autophagy process and some of its sub‐biological processes are significantly upregulated after Sunitinib treatment (Figure S4, Supporting Information). Moreover, the p62 gene SQSTM1 showed highest running enrichment score (ES = 0.51; Figure S4, Supporting Information) in GSEA analysis and significantly upregulated in sunitinib treatment group (Table S3, Supporting Information). To test the hypothesis that p62 regulates PD‐L1 via selective autophagy, we first investigated the colocalization of PD‐L1 with p62, LC3, or LAMP1. Confocal microscopic analysis indicated that cell surface located PD‐L1 showed negligible colocalization with p62, LC3, or LAMP1 in melanoma cells under the IFN‐*γ* induction. However, Sunitinib treatment accumulated PD‐L1 in cytoplasm and enhanced PD‐L1‐p62 and PD‐L1‐LC3/LAMP1 colocalization (**Figure** **5**A,B). This suggested that Sunitinib treatment promotes PD‐L1‐p62 interaction, which may subsequently induced the degradation of PD‐L1 in autophagosomes and lysosomes. Moreover, the co‐immunoprecipitation (IP) confirmed the interaction of PD‐L1 with p62 (Figure 5C,D). To substantiate these findings, we specifically knocked down p62 using a p62‐specific siRNA. The protein level of PD‐L1 did not change after Sunitinib treatment in the p62 knockdown group (Figure 5E). Similar results were also observed in triple‐negative breast cancer cells whose PD‐L1 expression is independent of IFN‐*γ* or in lung cancer cells which have a different type of IFN‐*γ* dependent PD‐L1 expression (Figure S5, Supporting Information). These results indicate that p62 is critical for selective autophagic degradation of PD‐L1.

### 2.4 Synergistic Effect of Sunitinib and CTLA‐4 mAb in the Treatment of Immune Competent Mice

**Figure 6:**
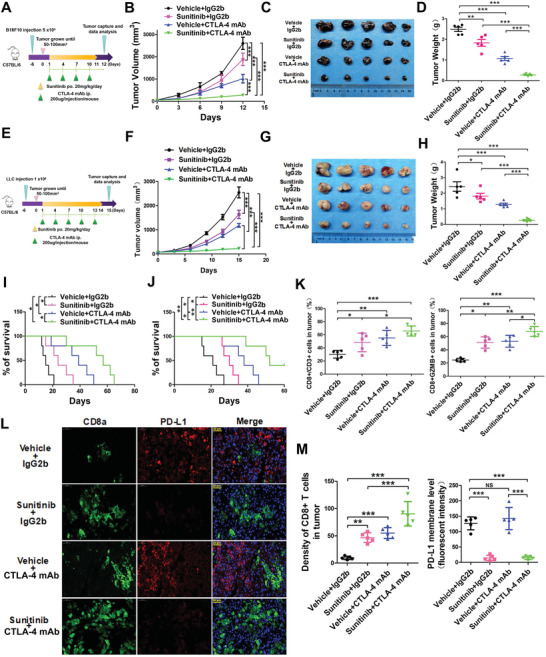
The combination of Sunitinib and CTLA‐4 mAb effectively suppresses melanoma tumor growth in vivo. A) Schematic diagram illustrating the treatment protocol of mAb and/or Sunitinib in B16F10 cells constructed mice model. At the endpoint, tumor cells and tumor‐infiltrating lymphocytes (TIL) were isolated for analysis. B) B16F10 tumor volume was measured at the indicated time points. *n* = 5 mice per group. Data represent mean ± SD, *p < 0.05, ***p < 0.001. Tumor growth analysis for Figure 2B through pairwise comparisons at each time in Table S1 in the Supporting Information. C) Representative images of B16F10 tumors after CTLA‐4 mAb and/or Sunitinib treatment at the end of time point in the B16F10 tumor burden mouse model. D) B16F10 tumor weight was measured at the endpoint, *n* = 5 mice per group. Data represent mean ± SD, *p < 0.05,***p < 0.001. E) Schematic diagram illustrating the treatment protocol of CTLA‐4 mAb and/or Sunitinib in LLC cells constructed mice model. At the endpoint, tumor cells and tumor‐infiltrating lymphocytes (TIL) were isolated for analysis. F) LLC tumor volume was measured at the indicated time points. *n* = 5 mice per group. Data represent mean ± SD, *p < 0.05, ***p < 0.001. Tumor growth analysis for Figure 2F through pairwise comparisons at each time in Table S1 in the Supporting Information. G) Representative images of LLC tumors after CTLA‐4 mAb and/or Sunitinib treatment at the end of time point in the LLC tumor burden mouse model. H) LLC tumor weight was measured at the endpoint, *n* = 5 mice per group. Data represent mean ± SD, *p < 0.05,***p < 0.001. I,J) Kaplan–Meier survival curves for mice bearing I) B16F10 and J) LLC tumors following treatment with CTLA‐4 mAb and/or Sunitinib. Significance was determined by the Gehan–Breslow–Wilcoxon test (*n* = 10 mice per group). Data represent mean ± SD, *p < 0.05,***p < 0.001. K) The quantification the CD8^+^/CD3^+^ and CD8^+^ GZMB^+^ CTL cell percentage in the B16F10 tumor mass from C57/BL6 mice treated with CTLA‐4 mAb and/or Sunitinib (*n* = 5 mice per group). Data represent mean ± SD, *p < 0.05,***p < 0.001. L) Representative image of immunofluorescence staining for immunostaining of CD8a and PD‐L1 in the B16F10 tumor mass treated with CTLA‐4 mAb and/or Sunitinib. The fluorescence of green (FITC) indicated the CD8 and red (Cy3) indicated the PD‐L1. Blue (DAPI) indicated the nucleus. Scale bar, 50 µm in inset. M) The quantification percentage of the CD8^+^ PD‐L1^+^ cell in the B16F10 tumor mass from mice treated with CTLA‐4 mAb and/or Sunitinib (*n* = 5 mice per group). Data represent mean ± SD, *p < 0.05,***p < 0.001.

Combination treatment targeting PD‐L1 and CTLA‐4 was utilized for cancer patients to improve the antitumor T‐cell immunity recently. Based on aforementioned results that Sunitinib can improve antitumor immunity by downregulating PD‐L1 signaling pathway, we hypothesized that Sunitinib may present similar effect as PD‐1 monoclonal antibody (mAb), which had synergistic effect with CTLA‐4 mAb in the treatment of immune competent mouse. To test this, B16F10 and Lewis lung cancer (LLC) tumor‐bearing mouse models were treated with Sunitinib, CTLA‐4 mAb, Sunitinib plus CTLA‐4 mAb, and control, respectively. As the results shown, in B16F10 melanoma model, Sunitinib alone significantly decreased tumor growth at day 12 after treatment compared to control group (mean tumor size: 1885 vs 2602 mm^3^; *p* < 0.001), while a combinatorial treatment of Sunitinib and anti‐CTLA‐4 reach the better efficacy (mean tumor size: 274 vs 2602 mm^3^; *p* < 0.001; **Figure** **6**A–D). Sunitinib alone substantially extended overall survival (median survival: 15 vs 24 d; *p* < 0.05) of B16F10 tumor‐bearing mice, and combined with anti‐CTLA‐4 enhanced survival benefit (median survival time: 15 vs 57 d; *p* < 0.05; Figure 6I). LLC lung cancer model also showed similar results, mice given the Sunitinib treatment inhibited tumor growth at day 15 after treatment compared to control group (1758 vs 2543 mm^3^; *p* < 0.001) and extend survival time (21 vs 30 d; *p* < 0.05), while a combinatorial treatment enhanced a decrease of tumor growth (225 vs 2543 mm^3^; *p* < 0.001; Figure 6E–H) and an extending of survival (21 vs 52 d; *p* < 0.01; Figure 6J). More importantly, when combined with CTLA‐4 mAb therapy, Sunitinib showed the best tumor growth‐inhibitive effect without side effects (Figure S6A,B, Supporting Information). Compared with the control or even monotherapies, the Sunitinib plus CTLA‐4 mAb group showed significantly increased survival rate and increasingly stronger antitumor effect along with treatment time, after adjusting for factors like body weight (Figure 6I–J and Table S1, Supporting Information). In consistent with our mechanistic findings, immunofluorescence and flow cytometric analysis showed that mono‐Sunitinib or Sunitinib plus CTLA‐4 mAb treatment significantly decreased PD‐L1 level, increased CD8^+^ CTL population, and enhanced their activity in the tumor region of immune competent mice (Figure 6K–M and Figure S6C–E, Supporting Information). Therefore, these findings suggest that Sunitinib is a potential combinatorial agent to enhance the efficacy of CTLA‐4 mAb therapy in the treatment of melanoma or NSCLC.

### 2.5 p62 Expression Level is Correlated with the Efficacy of PD‐L1 mAb Therapy in NSCLC Patients

**Figure 7:**
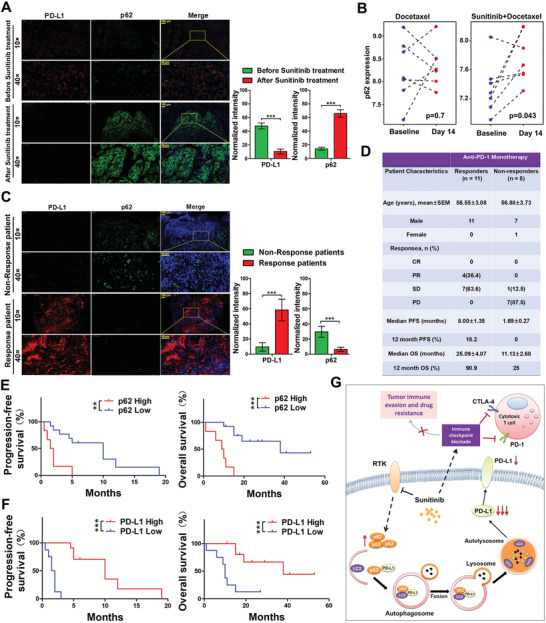
The reduction of PD‐L1 by Sunitinib induced p62 is clinically relevant. A) Representative image (left) and the quantification of normalized fluorescence intensity (right) of immunofluorescence staining for immunostaining of p62 and PD‐L1 in tumor mass from before and after Sunitinib treatment patients. The fluorescence of green (FITC) indicated the p62 and red (Cy3) indicated the PD‐L1. Blue (DAPI) indicated the nucleus. Scale bar, 50/100 µm in inset. The quantification of normalized fluorescence intensity was expressed as mean ± SEM, *n* = 6 views per sample, ***p < 0.001. B) Docetaxel‐ and sunitinib + docetaxel‐induced changes from baseline in the p62 expression to 14 d after treatment in cancer patients (*n* = 14). C) Representative image (left) and the quantification of normalized fluorescence intensity (right) of immunofluorescence staining for immunostaining of p62 and PD‐L1 in tumor mass from anti‐PD‐1 treatment response or nonresponse patients. The fluorescence of green (FITC) indicated the p62 and red (Cy3) indicated the PD‐L1. Blue (DAPI) indicated the nucleus. Scale bar, 50/100 µm in inset. The quantification of normalized fluorescence intensity was expressed as mean ± SEM, *n* = 10 samples per group, ***p < 0.001. D) Clinicopathologic characteristics of anti‐PD‐1 monotherapy cohorts. Anti‐PD‐1, antiprogrammed death‐1; CR, complete response; PR, partial response; SD, stable disease; PD, progressive disease; PFS, progression‐free survival; OS, overall survival. Patients were stratified into response groups based on RECIST (Response Evaluation Criteria in Solid Tumors) 1.1 criteria. Patients with CR, PR, and SD > 3 months were classified as responders, while patients with SD ≤ 3 months and PD were classified as nonresponders. E) Kaplan–Meier estimates for progression‐free survival and overall survival; patients (*n* = 19) were stratified in two groups: high p62 expression and low p62 expression. Significance was determined by the Gehan–Breslow–Wilcoxon test. Data represent mean ± SD, *p < 0.05,**p < 0.01. F) Kaplan–Meier estimates for progression‐free survival and overall survival; patients (*n* = 19) were stratified in two groups: high PD‐L1 expression and low PD‐L1 expression. Significance was determined by the log rank test. Data represent mean ± SD, *p < 0.05,**p < 0.01. G) A proposed model illustrating the regulation of PD‐L1 stability was p62‐mediated selective autophagy degradation.

To validate our findings that Sunitinib treatment decreases the PD‐L1 level through the p62‐mediated selective autophagy, we analyzed the p62 and PD‐L1 level by immunofluorescence staining in RCC patient samples before or after Sunitinib treatment. Compared to those of before Sunitinib treatment, we detected a higher level of p62 and a lower level of PD‐L1 in samples after Sunitinib treatment (**Figure** **7**A). Meanwhile, we found that patients enrolled in a phase III trial of metastatic breast cancer treated with docetaxel alone were no impact to the expression of p62; however, Sunitinib combined with docetaxel treatment significantly upregulated the p62 expression (Figure 7B). One reason that only a small subset of patients respond to PD‐1/PD‐L1 blockade is that the PD‐1 associated immune‐resistance depends on the accessibility of PD‐L1 ligand in the tumor. Furthermore, we analyzed the levels of p62 and PD‐L1 in the biopsy of 19 case NSCLC patients under anti‐PD‐1 (Nivolumab) monotherapy. As expected, a strong negative correlation of p62 and PD‐L1 was observed in all samples, in which the nonresponsive samples have a relatively higher p62 and a lower PD‐L1 signal compared with those of the responsive group (Figure 7C–D, **Table** **1**, and Table S4, Supporting Information). Moreover, we observed that patients with a low p62 expression in tumor region had a better PFS (progression‐free survival, median PFS 10 months vs 2 months, Gehan–Breslow–Wilcoxon test, *p*  =  0.0068) and an improved OS (*p*  =  0.0007, Figure 7E) after anti‐PD‐1 treatment. Consistent with previous literature, patients with a high PD‐L1 expression benefit from anti‐PD‐1 immunotherapy, as demonstrated by a significantly longer OS and PFS (Figure 7F). Taken together, our results revealed a novel molecular mechanism regarding the regulation of PD‐L1 stability was p62‐mediated selective autophagy degradation. We developed a potential therapeutic strategy for the treatment of melanoma and NSCLC patients by cotreatment of Sunitinib and CTLA4 mAb (Figure 7G).

**Table 1:** Correlations between p62 and PD‐L1 expression levels in NSCLC patients with anti‐PD‐1 mAb treatment

No. expression of PD‐L1^a)^ [%]					
		High	Low	Total	*P*‐value
p62	High	1	5	6	
	Low	10	3	13	
	Total	11	8	19	0.041

^a)^ *P*‐value by Fisher's exact test (two‐sided test). Low expression: positive rate <50%. High expression: positive rate ≥ 50%.

## 3 Discussion

Blockades targeted on PD‐1/PD‐L1 have been approved for treating human cancers with considerable clinical effects. However, the overall response rate to PD‐1/PD‐L1 blockades is relatively low, and the underlying mechanism is still unclear. Recent studies revealed that tumor PD‐L1 level is related to the efficacy of PD‐1/PD‐L1 blockades. Therefore, it is important to understand the molecular mechanism underlying the regulation of tumor PD‐L1. Sunitinib has been reported to have antitumor effect, but its role in cancer immunity is not well understood. Here we reported for the first time that Sunitinib improved OS in immune competent melanoma mouse model in vivo by induction of tumor CTL activity via alleviating tumor PD‐L1 expression level. Mechanistically, utilizing both in vitro and in vivo studies, we demonstrated that Sunitinib post‐translationally regulated PD‐L1 stability via p62‐dependent selective autophagy and confirmed this regulation in RCC patient samples. Preclinically, we showed that Sunitinib had synergistic effect with CTLA‐4 mAb in the treatment of melanoma and NSCLC immune competent mice. Clinically, we observed lower PD‐L1 levels and higher p62 levels in tumor region of nonresponders as compared to responders in anti‐PD‐1‐treated NSCLC lung cancer patients. Taken together, our studies revealed a novel mechanism regarding the regulation of PD‐L1, identified a potential prognostic marker for anti‐PD‐1 treatment efficacy, and provided a new combinatorial therapeutic strategy for the treatment of melanoma.

Sunitinib is FDA‐approved tyrosine kinase inhibitor, which was utilized to treat cancer patients in clinic.^[^ ^32^ ^]^ However, the role of Sunitinib in tumor immunity is still unclear. In this study, we found that Sunitinib suppressed protein level of tumor PD‐L1, therefore subsequently promoted CTL activity. To further test the clinical relevance of Sunitinib‐mediated inhibition of tumor PD‐L1 and subsequent immune surveillance, we utilized combination therapeutic strategy by cotreating Sunitinib and CTLA‐4 mAb in both melanoma and NSCLC immune competent mice. Intriguingly, we observed that Sunitinib had synergistic effect with CTLA‐4 mAb which significantly inhibited tumor growth and prolonged the OS rate by promoting immune surveillance. Sunitinib is a multitargeted RTK inhibitor targeting vascular endothelial growth factor receptor (VEGFR), platelet‐derived growth factor receptor (PDGFR), C‐KIT (CD117), REarranged during Transfection (RET), CSF1R, and (FMS‐like tyrosine kinase 3, (FLT‐3).^[^ ^33^ ^]^ These targets are highly conserved between human and mouse genome.^[^ ^34^ ^]^ Furthermore, recent study showed that Sunitinib treatment alone or in combination with anti‐VEGFR could enhance CD8+ T‐cell numbers in mouse models or in metastatic renal cell carcinoma (mRCC) patients,^[^ ^35^, ^36^ ^]^ suggesting the similar specificities in mice compared with human, and these correspondence between humans and mice reinforce the validity of mouse models for human diseases. Therefore, these results suggest that combination treatment of Sunitinib and CTLA‐4 mAb can be potential as a novel therapeutic strategy for the treatment of human melanoma and NSCLC.

Tumor PD‐L1 was reported to be regulated both at transcriptional and post‐transcriptional levels. A previous study reported that Sunitinib was characterized as an autophagy inducer, which was involved in specific substrate degradation.^[^ ^37^ ^]^ Here, we revealed that Sunitinib controlled PD‐L1 degradation by regulation of p62‐depended selective autophagy. By utilizing co‐immunoprecipitation and immunostaining, we demonstrated that PD‐L1 was interacted with p62 and colocalized in the lysosome. In summary, our data revealed that Sunitinib treatment regulated tumor PD‐L1 stability by induction of p62‐mediated selective autophagy. Mechanistically, this Sunitinib‐mediated downregulation of tumor PD‐L1 subsequently activates CTL activity, which promoted tumor surveillance. Preclinically, we demonstrated that combination treatment of Sunitinib and CTLA4 mAb significantly alleviated tumor burden and OS in melanoma and NSCLC immune competent mouse models.

## 4 Experimental Section

##### Cell Culture and Treatment

All cell lines used were obtained from American Type Culture Collection, Manassas, VA, USA. The human malignant melanoma cell lines (A375 and SK‐MEL‐28) and mouse LLC were cultured in dulbecco's modified eagle medium (DMEM) medium supplemented with 10% fetal bovine serum (FBS, Biological Industries), 100 U of penicillin, and 100 µg mL^−1^ streptomycin (Gibco). Human MDA‐MB‐231 breast cancer, human A549 lung cancer, and mouse melanoma cell line B16F10 were cultured in RPMI1640 medium. All cell lines were routinely tested for mycoplasma contamination and found to be negative.

Sunitinib was added to complete medium at the indicated concentrations and time. MG132 or BafA1 added to complete medium 6–12 h before harvest the cell.

##### Regents and Antibody

Anti‐SQSTM1/p62‐Rb (88588), LC3A/B (D3U4C) XP Rabbit mAb (12741) from Cell Signaling Technology; human anti‐PD‐L1 (ab213524 or ab213480); anti‐granzyme B antibody (ab4059) from Abcam; GAPDH (60004‐1‐Ig) from Proteintech; and anti‐CD8a (GB11068) from Servicebio were used. Sunitinib were purchased from Selleck (S1042), in vivo mAb antimouse CTLA‐4 (BE0164) and isotype antibody (BE0086) were purchased from Bioxcell. CY3 goat antirabbit IgG (GB21303), FITC goat antirabbit IgG (GB22303), and DAPI (G1012) CY5 goat antirabbit IgG were purchased from Servicebio.

##### RNA Isolation, Quantitative Real‐Time PCR

Total RNA was isolated from cultured human cancer cells using Invitrogen (TRIzol) according to the standard protocol. 1 µg total RNA was reverse‐transcribed using SuperScript III First‐Strand cDNA synthesis system (Life Technologies) according to the manufacturer's instructions Quantitative PCR was performed using a qPCR system (Eppendorf, Hamburg, Germany). All mRNA expression levels were normalized to GAPDH and calculated using the 2^−△△CT^ method. PD‐L1 primer forward sequences: TATGGTGGTGCCGACTACAA, Reverse sequences: TGCTTGTCCAGATGACTTCG; glyceraldehyde‐3‐phosphate dehydrogenase (GAPDH) primer forward sequence: CATGAGAAGTATGACAACAGCCT, Reverse sequences: AGTCCTTCCACGATACCAAAGT.

##### Western Blotting

Cells were lysed in cold RIPA buffer (Beyotime, China) in the presence of 1 × protease inhibitor cocktail and 1 × PhosStop (Roche, Isere, France) after two times phosphate buffer saline (PBS) washing. The viscosity of the lysate was removed by sonication, protein concentration was determined using a Pierce bicinchoninic acid (BCA) protein assay kit (Thermo Fisher Scientific, MA, USA). Equal amounts of proteins were loaded on to polyacrylamide gels.

##### Immunofluorescence

Cells were fixed with 4% paraformaldehyde, permeabilized in 0.1% Triton X‐100 (in phosphate buffer saline, PBS) and then blocked with bovine serum albumin (BSA). Slides were incubated with the indicated primary antibodies overnight, followed by incubation with FITC （fluorescein isothiocyanate） or Cy3‐conjugated secondary antibody for 1 h at room temperature (RT). The nuclei were stained with 4',6‐diamidino‐2‐phenylindole (DAPI), Sigma. For tumor sample, Cryostat sections were fixed with 4% paraformaldehyde for 15 min at RT. After PBS washing, incubate with 3% donkey serum, 1% BSA, 0.1% Triton X‐100 for 30 to 60 min at RT. Samples were stained with primary antibodies overnight at 4 °C, followed by FITC and/or Cy3 secondary antibodies at RT for 1 h. Hoechst 33342 (Life Technologies) was used for nuclear staining and images were visualized using a confocal microscope (Zeiss LSM 510, Germany).

##### Co‐Immunoprecipitation

For endogenous IP assays, cells were lysed in cold IP buffer (P0013, Beyotime, China) supplemented with protease‐inhibitor cocktail (Roche, France), 5–10% of the cell extract was saved as the input, and the rest was incubated with primary antibody at 4 °C overnight then add protein A/G agarose beads (Santa Cruz, USA) for 2–4 h at 4 °C. After three washes with the wash buffer (pH7.4 PBS with 0.1% Tiron‐X100), bound proteins were eluted by boiling with 2 × sodium dodecyl sulfate (SDS) loading buffer.

##### T Cell Mediated Killing Assay

To acquire activated T cells, human peripheral blood mononuclear cells (LTS1077, Yanjin Biological) were cultured in CTS AIIM V serum‐free medium (SFM) (A3021002; Gibco) with ImmunoCult Human CD3/CD28/CD2 T cell activator (10970; STEMCELL Technologies) and IL‐2 (1000 U mL^−1^; PeproTech, Rocky Hill, NJ, USA) for one week according to the manufacturer's protocol. The experiments were performed with anti‐CD3 antibody (100 ng mL^−1^; 16–0037; eBioscience, Thermo Scientific), interleukin‐2 (IL‐2), 1000 U mL^−1^. Cancer cells were seed in the plates overnight and then incubated Sunitinib (2.5 or 5 µmol) for 24 h, then incubate with activated T cells for 24 h. The ratios between cancer cells and activated cells were 1:3. T cells and cell debris were removed by PBS wash and left cells were quantified by a spectrometer at optical density (OD) 570 nm, followed by crystal violet staining.

##### Tumor Immune Cell Profile Analysis by Fluorescence activated Cell Sorting (FACS)

In this study, all flow cytometry antibodies and agents described above were purchased from BioLegend, San Diego, CA, USA. In mouse samples, single cell suspension of B16f10‐xenograft tumor was obtained by rapid and gentle stripping, physical grinding and filter filtration. After blocking with trustain fcX antimouse CD16/32 (101320) antibody and get rid of dead cells with Zombie Aqua Fixable Viability Kit (423102), cells were stained using APCCY7‐CD45 (103116), APC‐CD3(100236), PECY5.5‐CD4 (100434), PECY7‐CD8 (100722), BV421‐PD1 (135218) for 20 min. After fixation and permeabilization (421402), intracellular GZMB was stained using FITC‐GZMB (372206) antibody. To detect the expression of PD‐L1 on the membrane of human cell lines, cells were stained using PE‐PD‐L1 (329706), after blocking with Human TruStain FcX (422302) antibody and get rid of dead cells with Zombie Aqua Fixable Viability Kit (423102), all these antibodies were purchased from BioLegend. Stained cells were analyzed by FACS Dxp Athena and Aurora (Cytek, USA). Data were further analyzed by Flow Jo 10.0 software.

##### Mouse Tumor Generation and Implantation

All in vivo experiments were approved by the Animal Care and Use Committee of the third Xiangya Hospital of Central South University (Changsha, Hunan, China). Wlid‐type B16F10 cells (5 × 10^5^) were injected subcutaneously into 6‐week‐old C57BL/6 female mice (from the shanghai SLAC). Nearly one week later, mice were pooled and randomly divided into several groups. When observed the efficacy of Sunitinib alone, mice were treated daily with Sunitinib (20 or 40 mg kg^−1^, po) and vehicle control group. In the other part, the combined treatment effect of Sunitinib and checkpoint blockade was also observed. Wlid‐type B16F10 cells (5 × 10^5^) or LLC cells (1 × 10^6^) were injected subcutaneously into 6‐week‐old C57BL/6 female mice. Mice were treated with Sunitinib (20 mg kg^−1^, po), antimouse CTLA‐4 mAb (200 µg/mouse/3 d), combination therapy or single drug only, for 10 d. IgG2b isotype control and antimouse‐CTLA‐4 mAb (BE0164) treatments were conducted by intraperitoneal injection (200 µg per mouse in 100 µL D‐PBS buffer) every 3 d for a total of four to five injections. Subsequently, tumors were collected and analyzed by FACS. The removed xenografts were also snap‐frozen in liquid nitrogen. Paraffin embedded tumor blocks were prepared for further analysis at the same time.

##### Clinical Tissue Samples

Responders or nonresponders to Nivolumab (3 mg kg^−1^, q2w) treatment nonsmall cell lung carcinoma patients’ paraffin sections were collected from Hunan Cancer Hospital. The study protocol was approved by the Institutional Review Board of Hunan Cancer Hospital (SBQLL‐2019‐035). All tissue samples were collected in compliance with the informed consent policy. Clinical information is summarized in Table S4 in the Supporting Information.

##### RNA Sequencing Analysis

RNA sequencing was performed by Illumina technology to generate an average of 24 million raw paired‐end reads per sample. The mRNA polyA‐based enrichment method or rRNA depletion method was performed to establish RNA library. For differential gene expression analysis, the trimmed mean of M values method was used to normalize the expected gene counts data generated by RNA‐seq by expected maximization (RSEM). Only genes with at least 40 reads in total among all samples and at least 1 read in each of four samples were retained for normalization. This resulted in a total of 13687 genes used for downstream analysis. The normalized counts were fit into negative binomial generalized linear models (GLM) for differential expression analysis using edgeR,^[^ ^38^ ^]^ with tagwise dispersion and Sunitinib treatment as the univariate factor. Multiple testing was corrected by Benjamini–Hochberg procedure to control the false discovery rate (FDR) and to obtain the adjusted *p*‐values. Genes were considered to be differentially expressed if the fold‐change >1.3 and FDR < 0.05.

Gene ontology enrichment analysis was performed by clusterProfiler,^[^ ^39^ ^]^ where the differentially expressed genes identified as described above were supplied as the input for genes of interest and compare functional profiles by function compareCluster. Gene Set Enrichment Analysis^[^ ^40^ ^]^ (http://software.broadinstitute.org/gsea/index.jsp) was perform to test whether autophagy related gene sets are significantly enriched in the cell with Sunitinib treatment.

##### TCGA and Gene Expression Omnibus (GEO) Data Analysis

RNA expression data for patients enrolled in a phase III trial of metastatic breast cancer treated with Sunitinib combined with docetaxel versus docetaxel alone^[^ ^41^ ^]^ was downloaded from GEO (https://www.ncbi.nlm.nih.gov/geo/, GSE54323). INF‐*γ* related GO terms were download from The Molecular Signatures Database (MSigDB, http://software.broadinstitute.org/gsea/msigdb/).^[^ ^42^ ^]^ Immune checkpoint genes were obtained with known coinhibitory effects in T cells from a previous publication^[^ ^43^ ^]^ and 20 highly expressed checkpoints (median expression of RSEM > 5) were kept in at least one third of cancer types for the following analysis. Gene signature of suppressive immune cell populations was obtained from Charoentong et al.^[^ ^44^ ^]^ Immune “CYT” was calculated as the geometric mean of gene expression of cytolytic markers granzyme A (GZMA), perforin (PRF1).^[^ ^45^ ^]^ gene set variation analysis^[^ ^46^ ^]^ (GSVA) was used to calculate the score of IFN‐*γ* related GO terms and suppressive immune cell populations. GSVA score of IFN‐*γ* related GO terms were considered differential expression between baseline and 14 d after treated with Docetaxel or Sunitinib + Docetaxel paired samples if two‐sided paired Student's *t*‐test *p* < 0.05. The Spearman correlation between the expression of immune features and the Sunitinib targets was calculated, considering |Rs| > 0.3 and FDR < 0.05 for statistical significance.

##### Statistical Analysis

For the tumor growth data analysis, an overall difference at each data collection time point was tested by one‐way ANOVA. No experiments showed obvious bias toward a specific group in starting tumor volume. For comparisons among specific pairs of groups, statistical significance was assessed by the one‐way ANOVA followed by Tukey's multiple comparisons test. The assumption of ANOVA testing was checked to ensure the model assumption is not severely violated.

To evaluate the treatment efficacy, the linear mixed effect model (lme4 R package) was fitted by setting the individual mouse subject as the random effect, and body weight, days, treatment, and the interactions of days and treatment as the fixed effects. Model adequacy diagnostics were performed by checking the assumption of linearity, normality and homogeneity of variances of residuals generated by the model. Model fitting results were presented only when all assumptions are not violated.

For the animal survival data analysis, the log‐rank test was used to detect difference in Kaplan–Meier survival curves between groups. The relative risk of mouse death was computed by the Cox proportional hazard model where the treatment option was set as the single covariate and the proportional hazard assumption was checked by analyzing the Schoenfeld residuals against the transformed time. The fitting results were reported only when this assumption is not violated.

For all the remaining between‐group comparisons, the one‐way ANOVA and Dunnett's multiple comparison test or two‐tailed *t*‐test were used to determine the statistical differences. A *p*‐value of less than 0.05 was considered statistically significant. All data of bar graphs are presented as mean stable disease (SD, three independent experiments) and the results were analyzed using the SPSS 18.0 software package. Other analysis and data visualization were performed using R (http://www.r-project.org, version 3.5.1) or Graphpad Prism software (GraphPad Software, Inc., version 5.0).

##### Ethical Approval and Consent to Participate

Responders or nonresponders to Nivolumab (3 mg kg^−1^, q2w) treatment nonsmall cell lung carcinoma patients’ paraffin sections were collected from Hunan Cancer Hospital. The study protocol was approved by the Institutional Review Board of Hunan Cancer Hospital (SBQLL‐2019‐035). All tissue samples were collected in compliance with the informed consent policy.

## Conflict of Interest

The authors declare no conflict of interest.

## Supporting information

Supporting Information
