# Supplementary material for: The Beneficial Role of Sunitinib in Tumor Immune Surveillance by Regulating Tumor PD‐L1
Source: Adv Sci (Weinh). 2020 Nov 27;8(2):2001596. doi: 10.1002/advs.202001596 (PMC7816704; doi:10.1002/advs.202001596)
Supplement: Supplementary file 1 — Supporting Information [file ADVS-8-2001596-s001.pdf]

© 2020 Wiley-VCH GmbH

ADVANCED SCIENCE
Open Access

Supporting Information

for *Adv. Sci.,* DOI: 10.1002/advs.202001596

The beneficial role of Sunitinib in tumor immune surveillance by regulating tumor PD-L1

Hui Li, Xinwei Kuang, Long Liang, Youqiong Ye, YongChang Zhang, Jialu Li, Fangyu Ma, Juan Tao, Guang Lei, Shuang Zhao, Juan Su, Nong Yang, Cong Peng, Xiaowei Xu, Mien-Chie Hung, Leng Han, Hong Liu*, Jing Liu*, Xiang Chen**

The beneficial role of Sunitinib in tumor immune surveillance
by regulating tumor PD-L1

Hui Li[+], Xinwei Kuang[+], Long Liang[+], Youqiong Ye[+], YongChang Zhang, Jialu Li, Fangyu Ma, Juan Tao, Guang Lei, Shuang Zhao, Juan Su, Nong Yang, Cong Peng, Xiaowei Xu, Mien-Chie Hung, Leng Han, Hong Liu*, Jing Liu*, Xiang Chen**

Supplemental Table 1. Tumor growth analysis results for Figure 1C, Figure 4D and Supplemental Figure 4C. There are two tables for the data in each figure. The first table summarizes the pairwise comparison p-value at each time point, with a smaller p-value indicating a larger difference between the two groups. The second table presents the results of mixed-effect model fitting as described in Methods. The tumor volume data was log2 transformed before model fitting. For the fixed effect of treatment options, either the control or the combined therapy of interest (eg: Sunitinib +CTLA-4 mAb for Fig 4D) was set as the reference group. The significant interaction effect between time and treatment indicates that the treatment has a higher anti-tumor efficacy as time goes when compared to the control group.

**Pairwise Comparisons at each time point (Fig 1C)**	**0**	**3**	**6**	**9**	**12**
Su 20 vs. Control	1	0.6548	0.2268	0.0071	<0.001
Sun 40 vs. Control	1	0.5324	0.0296	0.0004	<0.001
Su 20 vs. Sun 40	1	0.9768	0.4696	0.2678	<0.001

**Treatment effect analysis Fig 1C**	**Estimate**	**P value**
**Days**	0.42613	<0.0001
**Sun 20**	-0.11767	0.62045

**Sun 40**	-0.04996	0.83400
**Weight**	-0.04009	0.43161
**Days: Sun 20**	-0.05058	<0.00942
**Days: Sun 40**	-0.13653	<0.001

**Days Comparisons (Fig 4D)**	**0**	**3**	**6**	**9**	**12**
Sunitinib+IgG2b vs. Sunitinib+CTLA-4 mAb	0.9954	0.2390	0.0112	<0.0001	<0.0001
Vehicle+CTLA-4 mAb vs. Sunitinib+CTLA-4 mAb	0.9992	0.2138	0.0247	0.0002	<0.0001
Vehicle+IgG2b vs. Sunitinib+CTLA-4 mAb	0.9972	<0.0001	<0.0001	<0.0001	<0.0001
Vehicle+CTLA-4 mAb vs. Sunitinib+IgG2b	0.9831	0.9999	0.9787	0.0442	<0.0001
Vehicle+IgG2b vs. Sunitinib+IgG2b	0.9729	0.0003	0.0225	0.0155	<0.0001
Vehicle+IgG2b vs. Vehicle+CTLA-4 mAb	0.9999	0.0004	0.0102	<0.0001	<0.0001

**Fig 4D**	**Estimate**	**P value**
**Days**	0.20728	<0.0001
**Sunitinib+IgG2b**	0.05255	0.78854
**Vehicle+CTLA-4 mAb**	0.28002	0.15591
**Vehicle+IgG2b**	0.61890	0.00226
**Weight**	-0.01268	0.63811
**Days: Sunitinib+IgG2b**	0.23544	<0.0001
**Days: Vehicle+CTLA-4 mAb**	0.15706	<0.0001
**Days: Vehicle+IgG2b**	0.23833	<0.0001

**Days Comparisons (Sup Fig 4C)**	**0**	**3**	**6**	**9**	**12**
Sunitinib+IgG2b vs. Sunitinib+CTLA-4 mAb	1.0000	0.4000	0.0248	<0.0001	0.0001
Vehicle+CTLA-4 mAb vs. Sunitinib+CTLA-4 mAb	0.9999	0.6185	0.1880	0.0016	0.0011
Vehicle+IgG2b vs. Sunitinib+CTLA-4 mAb	0.9976	0.0716	0.0001	<0.0001	<0.0001
Vehicle+CTLA-4 mAb vs. Sunitinib+IgG2b	0.9996	0.9802	0.6960	0.3005	0.4769
Vehicle+IgG2b vs. Sunitinib+IgG2b	0.9988	0.7180	0.0391	0.0002	0.0005
Vehicle+IgG2b vs. Vehicle+CTLA-4 mAb	0.9946	0.4926	0.0043	<0.0001	<0.0001

**Supplemental Fig 4C**	**Estimate**	**P value**
Days	0.09566	<0.0001
Sunitinib+IgG2b	-0.02342	0.9032
Vehicle+CTLA-4 mAb	-0.05995	0.7578
Vehicle+IgG2b	0.10332	0.5936
**Weight**	0.08644	0.0634
Days: Sunitinib+IgG2b	0.19625	<0.0001
Days: Vehicle+CTLA-4 mAb	0.17227	<0.0001
Days: Vehicle+IgG2b	0.24570	<0.0001

Supplemental Table 2. List of significantly differentially-expressed genes that belong to the biological process of selective autophagy.

Symbol	logFC	logCPM	FDR
SESN2	1.33843018	6.52706829	1.07E-44
OPTN	1.09085167	3.57344674	1.31E-09
SPTLC1	0.70923036	4.94083565	3.38E-08
BECN1	0.59320344	5.33663922	1.26E-07
CALCOCO2	0.69310023	4.48507248	6.76E-07
MFN2	-0.3981555	8.36942453	8.76E-07
RNF41	0.38443553	7.29518992	3.95E-06
SQSTM1	**0.33166395**	**10.6534671**	**7.24E-05**
ADRB2	-0.8594578	2.88621709	0.00013687
HDAC6	0.42672627	5.41339149	0.00018645
HTT	-0.308237	7.0191931	0.00043588
MAPK3	-0.3207781	7.05625063	0.00072537
WIPI2	-0.2846679	7.44758998	0.00106038
HTRA2	-0.2760406	6.7645145	0.00221018
LGALS8	0.48193938	4.16776755	0.00231538
ATG14	0.59298676	3.49286434	0.00294681
TOMM7	0.4029165	5.55853603	0.00870344
WDFY3	0.69091522	2.38409663	0.01245925
RETREG1	1.27981231	0.38610981	0.01706579
SMURF1	-0.2553462	5.78289634	0.01842524
UBQLN1	0.22730394	6.32636441	0.02312429
VPS13C	0.7992966	1.23922786	0.0409454
NOD1	-0.3949972	3.68872132	0.04170285
ATG16L1	-0.2580434	5.00188992	0.04598677
AMBRA1	-0.2022006	6.21618481	0.04808278

SQSTM1 is the p62 as described in the manuscript. logCPM represents the gene's relative expression level averaged across samples. Abbreviations: logFC, log fold change; logCPM, log counts per million; FDR, adjusted p-value after controlling for false discovery rate.

Supplemental Table 3. Clinicopathologic characteristics of anti-PD-1 monotherapy cohorts.

Patient No.	Response	Gender	Age	Tumor types	TNM stage	PS score	PFS (month)	OS (month)	Adverse effect	Metastatic lesion
1	PR	Male	68	LUAD	T2N3M1B	1	19	53+	None	Mediastinum, axilla
2	SD	Male	50	LUAD	T2N3M1B	1	10	38+	None	Pleura
3	SD	Male	50	LUAD	T3N3M1	1	6+	17+	None	Bone liver adrenal gland
4	SD	Male	40	LUAD	T4N3M1	1	6+	41+	erythra	pleura, multiple bones
5	PR	Male	61	LUSC	T1N3M1B	1	6+	15+	I° hepatic damage	Bone
6	PR	Male	57	LUSC	T2N0M1	1	4.5+	23+	I° hypothyroidism	Bone
7	SD	Male	59	LUSC	T2N2M1	1	12	15	I° hepatic damage	Bone
8	SD	Male	66	LUSC	T4N2M1	1	4.5	11+	None	brain, adrenal gland
9	PR	Male	50	LUSC	T2N3M0	1	10	29+	epilepsy	Iliac crest, retroperitoneal lymph nodes
10	SD	Male	74	LUSC	T2N2M1	1	5	19	None	lung, liver
11	SD	Male	47	LUSC	T4N2M03 B	1	5	15	None	lung, mediastinal lymph nodes
12	PD	Male	57	LUAD	T2N2M1B	1	1.5	11	I° weak, cough	Bone
13	PD	Male	38	LUAD	T1N3M1B	1	2	9	I° emesis	Bone, adrenal, brain
14	SD	Male	63	LUAD	T1N0M1	1	3	10	None	lung, abdominal subcutaneous
15	PD	Female	54	LUAD	T3N0M1B	1	2	6	None	lung, bone
16	PD	Male	72	LUSC	T1N3M1	1	2	27+	arrhythmia	axilla, gluteus maximus, humerus
17	PD	Male	62	LUSC	T1N2M1	1	1	1	None	Bone
18	PD	Male	62	LUSC	T3N3M0	1	0.5	10	None	None
19	PD	Male	47	LUSC	T3N2M1a	1	1.5	15	II° hepatic damage	Pleura

Anti-PD-1, anti-programmed death-1; CR, complete response; PR, partial response; SD, stable disease; PD, progressive disease; PFS, progression-free survival; OS, overall survival.Patients were stratified into response groups based on RECIST 1.1 criteria. Patients with CR, PR, and SD > 3 months were classified as responders, while patients with SD≤3 months and PD were classified as non-responders. LUSC: Lung squamous cell carcinoma. LUAD: Lung adenocarcinoma. TNM stage based on The 8th Edition Lung CancerStage Classification.

Supplemental Figures

Supplemental Figure 1

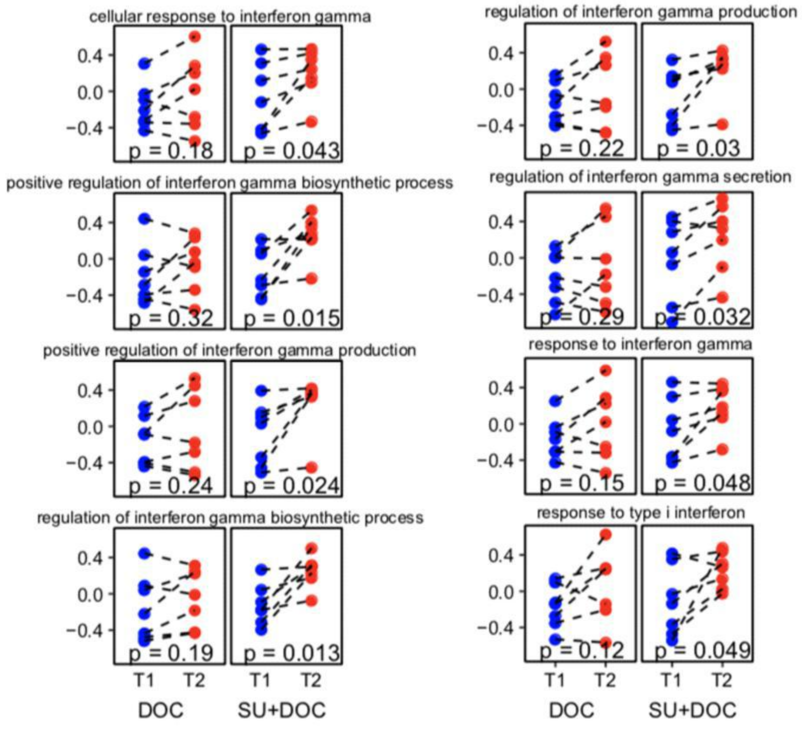

Supplemental Figure 1. Docetaxel- and sunitinib + docetaxel-induced changes from baseline in the GSVA of INFγ related GO terms to 14 days after treatment in cancer patients.

Supplemental Figure 2

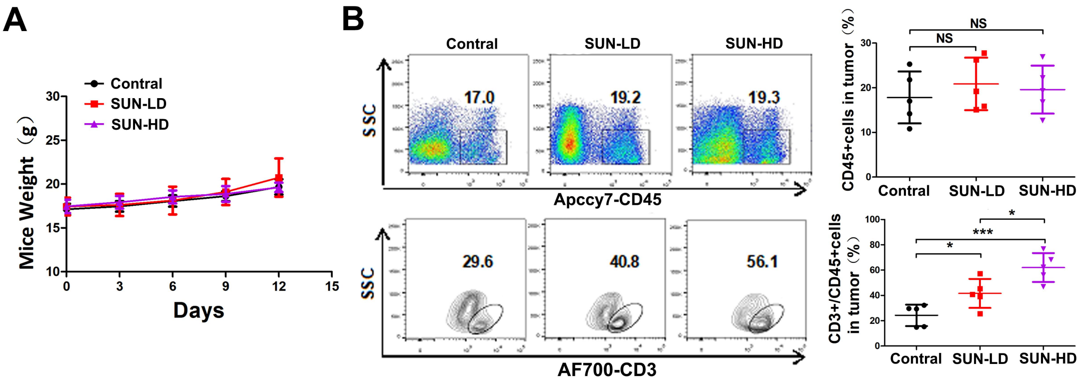

Supplemental Figure 2. Sunitinib increase the CTL population and no effect on mice body weight.

A. During the treatment period, mouse body weight was measured on the indicated time points Data are shown as mean±SD.

B. Representative profiles and the quantification of flow cytometry-based detection of CD45+CD3+ CTL in the tumor mass form Sunitinib treatment or vehicle. n=5 mice per group, data represent mean±SD, *p<0.05,***p<0.001.

Supplemental Figure 3

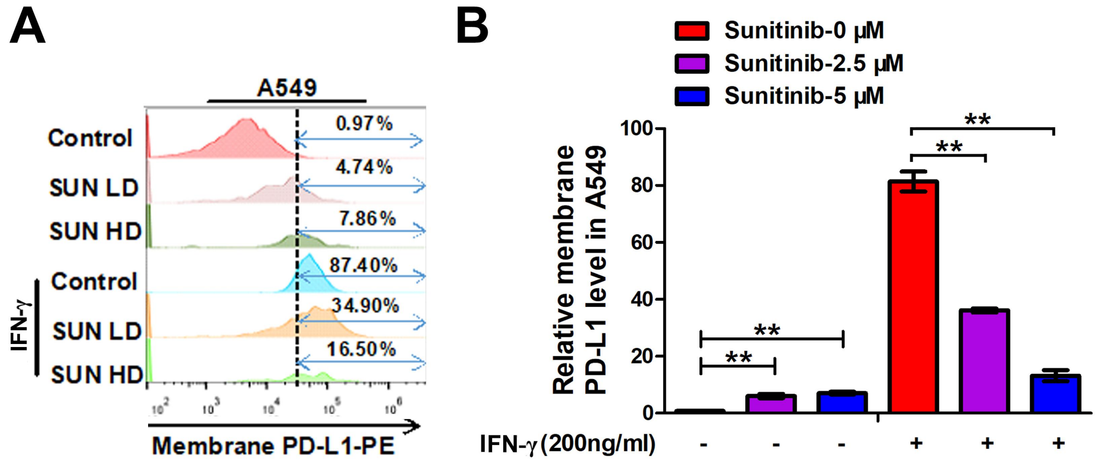

Supplemental Figure 3. Sunitinib exposure decreased membrane-located PD-L1 level in melanoma cells and A549 cells.

A. Representative profiles of flow cytometry analysis of membrane PD-L1 expression by flow-cytometric analysis after increasing concentrations of Sunitinib (2.5 to 5 μM) treated A549 cells for 24 hours under IFN-γ exposure.

B. The quantitative analysis of flow cytometry analysis of membrane PD-L1 expression by flow-cytometric analysis after increasing concentrations of Sunitinib (2.5 to 5 μM) treated A549 cells for 24 hours under IFN-γ exposure.

Supplemental Figure 4

A

	Term	Ont	N	Up	Down	P.Up	P.Down
GO:0044248	cellular catabolic process	BP	1761	488	390	4.784137e-08	0.9996886
GO:0009056	catabolic process	BP	1940	518	443	3.493355e-06	0.9961663
GO:0006914	autophagy	BP	410	130	90	1.633475e-05	0.9528864
GO:0061919	process utilizing autophagic mechanism	BP	410	130	90	1.633475e-05	0.9528864
GO:0050662	coenzyme binding	MF	220	76	46	1.686749e-05	0.9194302
GO:0016236	macroautophagy	BP	259	88	52	2.153604e-05	0.9803426

B

	Term	Ont	N	Up	Down	P.Up	P.Down	significance
GO:0006914	autophagy	BP	410	130	90	1.633475e-05	0.9528864	TRUE
GO:0061919	process utilizing autophagic mechanism	BP	410	130	90	1.633475e-05	0.9528864	TRUE
GO:0016236	macroautophagy	BP	259	88	52	2.153604e-05	0.9803426	TRUE
GO:0010506	regulation of autophagy	BP	264	79	59	4.018038e-03	0.8811540	TRUE
GO:0044754	autolysosome	CC	8	6	1	5.432286e-03	0.9353320	TRUE
GO:0005776	autophagosome	CC	79	28	11	7.537421e-03	0.9956572	TRUE
GO:0000045	autophagosome assembly	BP	84	28	15	9.572792e-03	0.9355934	TRUE
GO:1905037	autophagosome organization	BP	87	29	15	9.741947e-03	0.9588765	TRUE
GO:0016240	autophagosome membrane docking	BP	4	3	0	2.439379e-02	1.0000000	TRUE
GO:0016241	regulation of macroautophagy	BP	145	44	32	2.544329e-02	0.8576885	TRUE
GO:0030242	autophagy of peroxisome	BP	5	3	0	2.778880e-02	1.0000000	TRUE
GO:0032258	protein localization by the Cvt pathway	BP	2	2	0	3.698963e-02	1.0000000	TRUE
GO:0097352	autophagosome maturation	BP	35	13	6	4.425284e-02	0.9176229	TRUE
GO:0061912	selective autophagy	BP	43	15	10	4.891990e-02	0.6791124	TRUE

Supplemental Figure 4. Sunitinib induce autophagy in melanoma cells.

A. Top up-regulated GO terms identified from gene ontology analysis using the RNA sequencing data. Note that autophagy is a sub-GO term of catabolic process and cellular catabolic process.

B. List of significantly enriched autophagy-related biological processes or cellular components from the RNA sequencing data analysis.

Supplemental Figure 5

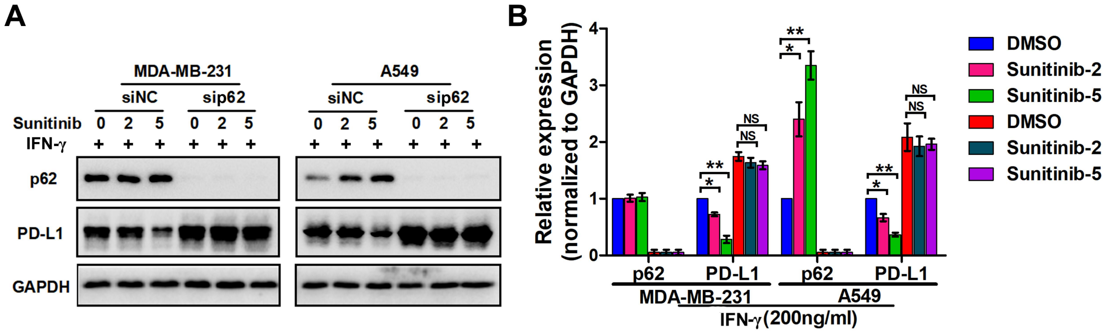

Supplemental Figure 5. PD-L1 decreased by Sunitinib induced p62 in breast and lung cancer cell line.

A. Representative western blot analysis of knockdown p62 in MDA-MB-231 (triple negative breast caner cell line) and A549 (lung cancer cell line) cells treated with increasing concentrations of Sunitinib (0 to 5 μM) for 24 hr under IFN-γ exposure.

B. Bar diagram presenting the quantitative analysis of protein expression data from A The plot was generated from three independent experiments and showed as means±SD (**p<0.01, *p<0.05).

Supplemental Figure 6

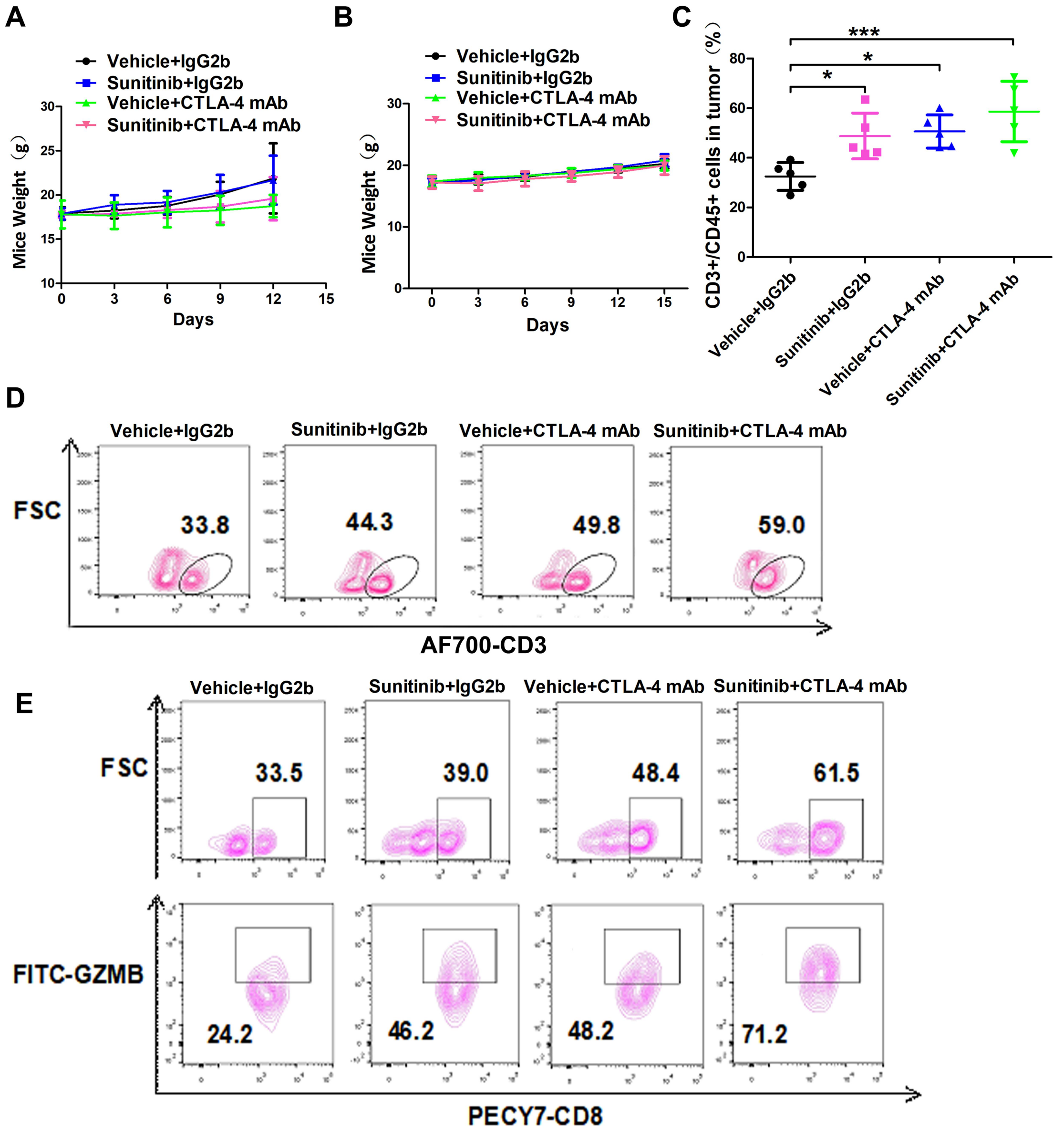

Supplemental Figure 6. The combination of Sunitinib and anti-CTLA-4 effectively suppresses NSCLC tumor growth *in vivo*

A. The mice body weight of B16F10 tumor model was measured at the indicated time points. n=5 mice per group. Data represent mean±SD, $^{NS}p>0.05$.

B. The mice body weight of LLC tumor model was measured at the indicated time points. n=5 mice per group. Data represent mean±SD, $^{NS}p>0.05$.

C. The quantification of flow cytometry-based detection of CD45+/CD3+ CTL in the tumor mass from C57/BL6 mice treated with CTLA-4 antibody and/or Sunitinib. n=5 mice per group, data represent mean±SD, *$p<0.05$,***$p<0.001$.

D. Representative profiles of flow cytometry-based detection of CD45+ CD3+ CD4+ CD8+ CTL in LLC tumor mass from C57/BL6 mice treated with CTLA-4 antibody and/or Sunitinib.

E. Representative profiles of flow cytometry-based detection of The CD8 (CTL marker) and granzyme B (GZMB) the marker of activity of the T cell in LLC tumor mass from C57/BL6 mice treated with CTLA-4 antibody and/or Sunitinib.
